# Multi-omics analysis reveals regime shifts in the gastrointestinal ecosystem in chickens following anticoccidial vaccination and *Eimeria tenella* challenge

**DOI:** 10.1128/msystems.00947-24

**Published:** 2024-09-17

**Authors:** Po-Yu Liu, Janie Liaw, Francesca Soutter, José Jaramillo Ortiz, Fiona M. Tomley, Dirk Werling, Ozan Gundogdu, Damer P. Blake, Dong Xia

**Affiliations:** 1 Pathobiology and Population Sciences, Royal Veterinary College, London, United Kingdom; 2 School of Medicine, College of Medicine, National Sun Yat-sen University, Kaohsiung, Taiwan; 3 Department of Biomedical Science and Environmental Biology, Kaohsiung Medical University, Kaohsiung, Taiwan; 4 Faculty of Infectious and Tropical Diseases, London School of Hygiene & Tropical Medicine, London, United Kingdom; 5 Scotland’s Rural College, Edinburgh, United Kingdom; 6 Centre for Vaccinology and Regenerative Medicine, Royal Veterinary College, London, United Kingdom; The University of Maine, Orono, Maine, USA

**Keywords:** *Eimeria*, yeast-based anticoccidial vaccine, gut microbiota, metabolome, multi-omics

## Abstract

Coccidiosis, caused by *Eimeria* parasites, significantly impacts poultry farm economics and animal welfare. Beyond its direct impact on health, *Eimeria* infection disrupts enteric microbial populations leading to dysbiosis and increases vulnerability to secondary diseases such as necrotic enteritis, caused by *Clostridium perfringens*. The impact of *Eimeria* infection or anticoccidial vaccination on host gastrointestinal phenotypes and enteric microbiota remains understudied. In this study, the metabolomic profiles and microbiota composition of chicken caecal tissue and contents were evaluated concurrently during a controlled experimental vaccination and challenge trial. Cobb500 broilers were vaccinated with a *Saccharomyces cerevisiae*-vectored anticoccidial vaccine and challenged with 15,000 *Eimeria tenella* oocysts. Assessment of caecal pathology and quantification of parasite load revealed correlations with alterations to caecal microbiota and caecal metabolome linked to infection and vaccination status. Infection heightened microbiota richness with increases in potentially pathogenic species, while vaccination elevated beneficial *Bifidobacterium*. Using a multi-omics factor analysis, data on caecal microbiota and metabolome were integrated and distinct profiles for healthy, infected, and recovering chickens were identified. Healthy and recovering chickens exhibited higher vitamin B metabolism linked to short-chain fatty acid-producing bacteria, whereas essential amino acid and cell membrane lipid metabolisms were prominent in infected and vaccinated chickens. Notably, vaccinated chickens showed distinct metabolites related to the enrichment of sphingolipids, important components of nerve cells and cell membranes. Our integrated multi-omics model revealed latent biomarkers indicative of vaccination and infection status, offering potential tools for diagnosing infection, monitoring vaccination efficacy, and guiding the development of novel treatments or controls.

**IMPORTANCE:**

Advances in anticoccidial vaccines have garnered significant attention in poultry health management. However, the intricacies of vaccine-induced alterations in the chicken gut microbiome and its subsequent impact on host metabolism remain inadequately explored. This study delves into the metabolic and microbiotic shifts in chickens post-vaccination, employing a multi-omics integration analysis. Our findings highlight a notable synergy between the microbiome composition and host-microbe interacted metabolic pathways in vaccinated chickens, differentiating them from infected or non-vaccinated cohorts. These insights pave the way for more targeted and efficient approaches in poultry disease control, enhancing both the efficacy of vaccines and the overall health of poultry populations.

## INTRODUCTION

Protozoan parasites of the genus *Eimeria* cause coccidiosis in poultry, and costs to the industry have been estimated to exceed £10 billion annually (1). Clinical coccidiosis manifests as poor body weight gain and feed conversion with diarrhea, bloody droppings, and mortality in severe cases. Infection induces strong pro- and anti-inflammatory cytokine responses that may exacerbate pathology (2–5). Clinical coccidiosis is commonly avoided through a combination of good husbandry, parasite chemoprophylaxis with anticoccidial drugs and/or vaccination using varied formulations of live parasites (6, 7). In some countries, public concern related to pathogen drug resistance and widespread use of antimicrobials in animal production are driving legislative and commercial changes, including increased use of anticoccidial vaccination (8). Although current live parasite vaccines are effective, considerable efforts are also being made to develop recombinant anticoccidial vaccines (9). In a previous study, a prototype inactivated yeast-based recombinant oral vaccine for *Eimeria tenella* was shown to result in reduced parasite replication, reduced caecal pathology and improved chicken performance compared to controls in commercial chickens (10). Using *Saccharomyces cerevisiae* to express and deliver *E. tenella* antigens apical membrane antigen 1 (EtAMA1) (11), immune mapped protein 1 (EtIMP1) (12), and repeat 3 from microneme protein 3 (EtMIC3) (13) induced significant protection against high-level challenge in vaccinated Cobb500 broiler chickens (10). However, the impact of vaccination and subsequent parasite challenge on the host gut and its enteric microbiota were not evaluated. Oral administration of heat-inactivated and freeze-dried *S. cerevisiae* has previously been shown to ameliorate the effects of coccidiosis in broiler chickens while modulating the host immune response and microbiota (14, 15). Understanding the influence of a yeast-vectored anticoccidial vaccine on host-microbe interacted metabolome and microbiomes could therefore be used to improve future vaccine development.

Enteric microbiomes play crucial roles in shaping host physiological functions including provision of nutrients (16, 17), immune system maturation, and regulation (18, 19). *Eimeria* infection can cause imbalance in gastrointestinal (GI) ecosystems (20, 21), commonly referred to as dysbiosis, and raises the risk of enteric comorbidities such as necrotic enteritis caused by *Clostridium perfringens* (22). Variation in the severity of damage caused by *Eimeria* infection has also been shown to be associated with differences in enteric microbiomes. For example, high-level caecal lesion scores recorded during *E. tenella* infection correlated with increased *Enterobacteriaceae* occurrence but decreased *Bacillales* and *Lactobacillales* (21). However, little is known about physiological responses in gastrointestinal molecular and biochemical mechanisms, or variation in microbiota between immunologically naïve, infected, and vaccinated chickens. Few studies have provided insight into chickens' metabolic responses to infection or vaccination. Using an untargeted metabolomic profile assessment, Aggrey et al. (23) found that carnitine-derived metabolites involved in fatty acid metabolism, and thromboxane B2, 12-HHTrE, and itaconate involved in inflammatory responses, were influenced by *Eimeria acervulina* infection (23). In the same way, a human shingles vaccine trial revealed that key metabolites such as sterol class metabolites, arachidonic acids, phosphoinositide, and diacylglycerol, were essential to immune signaling (24). Here, we have created a multi-omics data set defining caecal microbial populations (lumen contents and tissue-associated) and caecal tissue metabolomes using high-throughput sequencing of the 16S rDNA and liquid chromatography-mass spectrometry (LC-MS), respectively. We have used a multi-omics factor analysis (MOFA) (25, 26) machine learning model to systematically integrate data on caecal microbiota and the caecal metabolome sampled during an anticoccidial vaccine trial, investigating host microbe-associated signatures that can predict chicken health status and vaccine efficacy.

## RESULTS

### Caecal pathology and parasite load post-*Eimeria* challenge demonstrates efficacy of a candidate yeast-vectored anticoccidial vaccine in commercial broiler chickens

**Fig 1:**
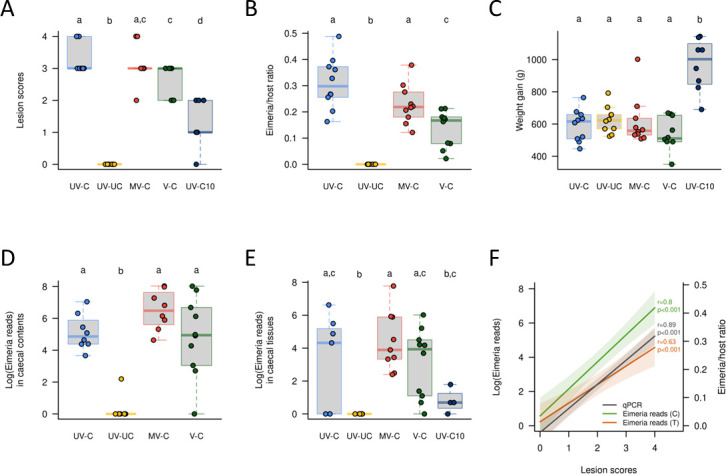
Summary of vaccine trial phenotypes assessed 6 days post-infection (dpi) with 15,000 sporulated *Eimeria tenella* oocysts. (**A**) Caecal lesion scores, (**B**) parasite load represented as parasite genomes per host genome, determined using qPCR, (**C**) body weight gain from 0 to 6 dpi, (**D and E**) parasite load represented by *Eimeria* apicoplast 16S rDNA sequence reads in caecal contents and tissue, (**F**) association between caecal lesion score and parasite load measures. Panels (A–C) reanalyzed by Soutter et al. (10). Groups UV-C: unvaccinated, challenged, UV-UC: unvaccinated, unchallenged, MV-C: mock vaccinated, challenged, V-C vaccinated, challenged.

We previously evaluated the efficacy of an experimental *S. cerevisiae*-vectored anticoccidial vaccine using readouts of gut pathology (caecal lesion scores: 0–4), parasite replication (quantitative PCR of caecal tissue), and chicken performance (body weight gain, BWG) following oral challenge of Cobb500 broiler chickens reared under commercial conditions with 15,000 sporulated oocysts of *E. tenella* (10). Briefly, lesion scores at 6 days post-infection (dpi) were lower in vaccinated chickens compared to unvaccinated controls (V-C vs. UV-C; *P* < 0.001; Fig. 1A). Parasite replication measured by qPCR as parasite genomes per host genome was also lower in vaccinated chickens at 6 dpi (*P* < 0.001; Fig. 1B). In contrast, BWG was not significantly different at 6 dpi (Fig. 1C), although it was by 10 dpi (10).

In the present study, the level of *E. tenella* replication at 6 dpi was confirmed by quantification of *Eimeria* apicoplast 16S rDNA amplicon reads in NGS microbiome data from caecal tissue and contents (Fig. 1D and E). Comparison of all three *E. tenella* replication measures revealed a significant association with lesion score severity (qPCR ratio: *r* = 0.89, NGS 16S reads of caecal contents: *r* = 0.8, NGS 16S reads of caecal tissue: *r* = 0.63; all *P* < 0.001; Fig. 1F). For comparison, 10 dpi unvaccinated and challenged chickens (UV-C10) considered to be recovering from infection also showed a significant reduction in gut pathology and *Eimeria* load compared to all infected subjects at 6 dpi (*P* < 0.001; Fig. 1A and E).

### Gut pathology and parasite load correlate with changes in gut microbiota

The composition of enteric microbial populations can reflect the health status of microecosystems in the GI tract. We performed 16S rDNA amplicon sequencing from caecal contents and tissues collected from the same individuals to characterize gut microbiota composition, with no significant differences in beta diversity detected between sample types (caecal tissue compared to caecal contents; PERMANOVA test *R*^2^ = 0.026, *P* = 0.052) (Fig. S1A). Comparison between caecal contents and tissue found 62.7% to 73.6% of microbiota composition to be shared (Fig. S1B). Microbial populations enriched in caecal contents included *Lactobacillus mucosae*, *Lactobacillus salivarius*, *Paludicola psychrotolerans*, *Kineothrix alysoides*, *Anaerostipes butyraticus*, and [*Clostridium*] *polysaccharolyticum*; while microbial populations of *Anaerotruncus colihominis* (KTU 13) and *Flavonifractor plautii* (KTU 14) were enriched in caecal tissues (i.e., UV-C, MV-C, and V-C) (Fig. S1C).

**Fig 2:**
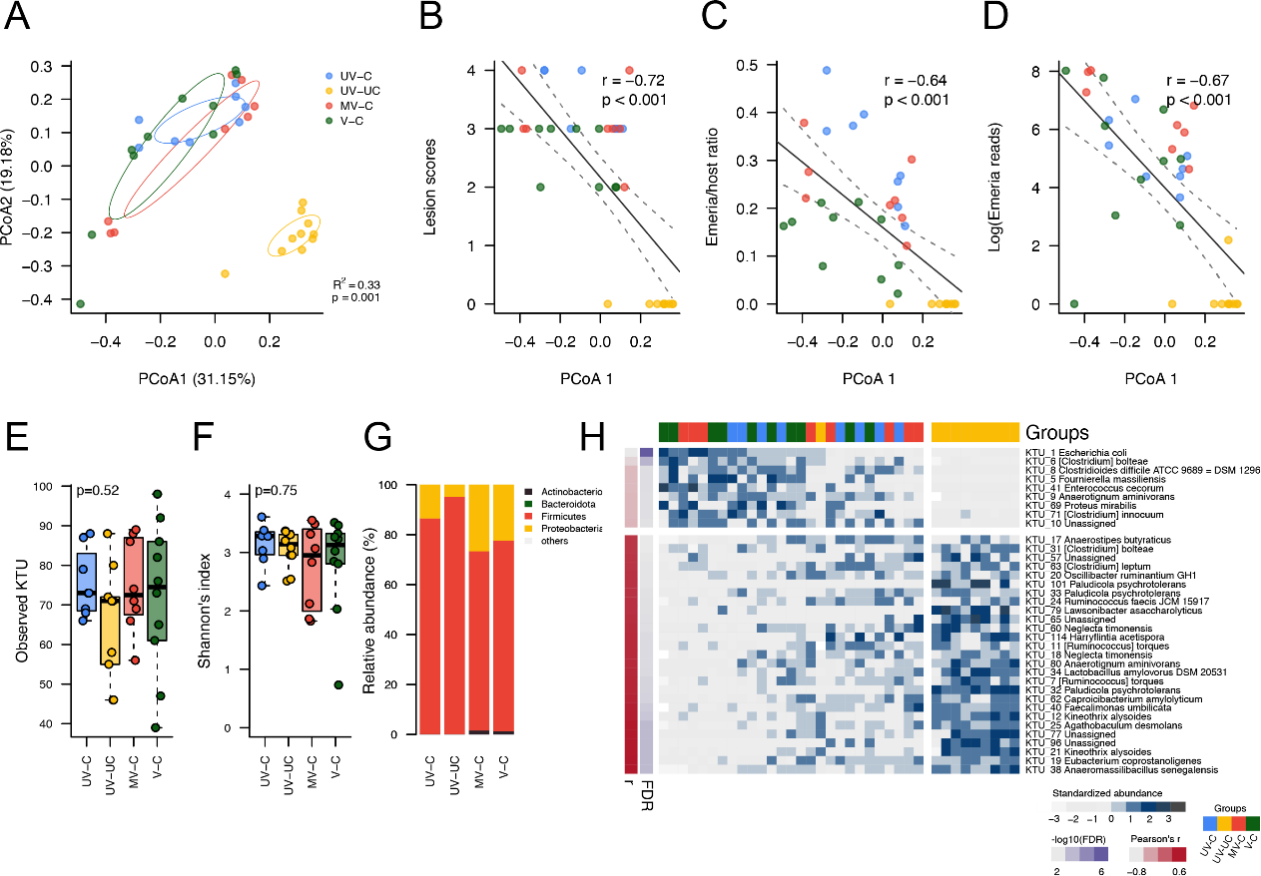
Gut microbiota profiling and associations of gut pathology and parasite loads. (**A**) Principal coordinates analysis (based on Bray-Curtis distance) for beta diversity of gut microbiota composition (caecal contents) among four groups of chickens. (**B**) The correlation between microbiota composition and lesion scores. (**C**) and (**D**) The correlations between microbiota composition and parasite loads, (**C**) based on qPCR quantification of the ratio of *Eimeria* and host genes, (**D**) based on NGS reads of *Eimeria* apicoplast 16S rDNA. (**E**) Alpha diversity (observed KTUs) of four groups of chickens. (**F**) Alpha diversity (Shannon’s index) of four groups of chickens. (**G**) Relative abundance of gut microbiota composition at the phylum level. (**H**) Gut pathology and parasite load-associated microbes (Pearson’s *r* > 0.4 or <−0.4, FDR-adjusted *P* < 0.05) extracted from PCoA1 of panel (**A**). Groups UV-C: unvaccinated, challenged, UV-UC: unvaccinated, unchallenged, MV-C: mock vaccinated, challenged, V-C vaccinated, challenged.

Principal coordinates analysis (PCoA) based on Bray-Curtis dissimilarity measurements showed that the caecal contents microbiota composition of unchallenged versus all challenged groups were distinct from each other (6 dpi) along the PCoA1 axis (31.15% of observed variation) (Fig. 2). A PERMANOVA test confirmed significant differences in microbiota (*R*^2^ = 0.33, *P* = 0.001) (Fig. 2A) and there were significant correlations with caecal lesion scores (|*r*| = 0.73, *P* < 0.001), parasite load in caecal tissues (qPCR ratio: |*r*| = 0.76, *P* < 0.001) and caecal contents (NGS reads: |*r*| = 0.67, *P* < 0.001) (Fig. 2B through D).

On average a low alpha diversity index of microbial richness (observed KTUs) was found in all chickens across all groups (71.64 ± 14.21; Fig. 2E) compared to a previous study by Hay et al. (27) (493.13 ± 201.60, reanalyzed using the same pipeline used in the present study) (27). This disparity may be due to the requirement for broad-spectrum enrofloxacin treatment during this trial, a common feature of commercial production systems, with no effect on Shannon’s diversity index (Fig. 2F). Comparison between the groups revealed higher observed KTUs in all challenged groups 6 dpi compared to the unvaccinated, unchallenged group (UV-UC), although the difference was not statistically significant. The dominant phyla were *Firmicutes*, followed by *Proteobacteria* in all chickens (combined, accounting for more than 98%) (Fig. 2G); however, *Proteobacteria* were reduced in UV-UC chickens (4.83% compared to 13.5%/26.65%/22.36% in other groups). *Actinobacteria* were enriched in both mock and true vaccinated groups (1.55% and 1.35%, respectively), dominated by genus *Bifidobacterium* (1.50% and 1.30%, respectively). Since the lesion scores and *Eimeria* loads were significantly correlated with the PCoA1 axis of beta diversity, 36 associated taxa enriched in challenged chickens were identified by Pearson’s correlation analysis [|*r*| ≥ 0.4, false discovery rate (FDR) < 0.1], including *Escherichia coli*, *Clostridium difficile*, *C. innocuum*, and *Proteus mirabilis* (Fig. 2H).

### Metabolomes reflect the molecular alterations of host physiology responses in health, infection, and recovery

**Fig 3:**
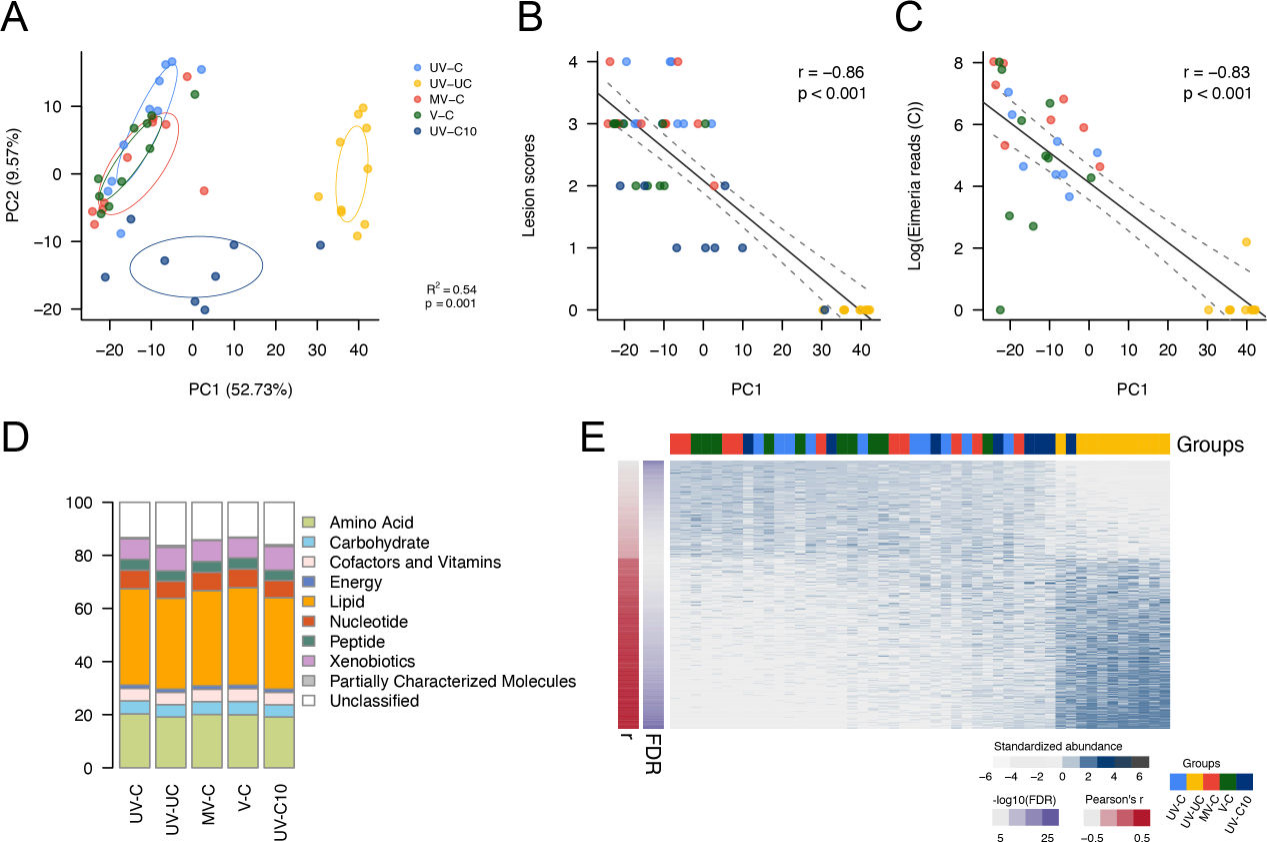
Chicken caecal tissue metabolome profiling and associations of gut pathology and parasite loads. (**A**) Principal component analysis for chicken caecal tissue metabolome composition among five groups of chickens. (**B**) The correlation between metabolome composition and lesion scores. (**C**) The correlation between metabolome composition and parasite loads, based on NGS reads of *Eimeria* apicoplast 16S rDNA. (**D**) Compositions and categories of the metabolome of five groups of chickens. (**E**) Gut pathology and parasite load-associated metabolites (Pearson’s *r* > 0.4 or <−0.4, FDR-adjusted *P* < 0.05) extracted from PC1 of panel (**A**). Groups UV-C: unvaccinated, challenged, UV-UC: unvaccinated, unchallenged, MV-C: mock vaccinated, challenged, V-C vaccinated, challenged, UV-C10: unvaccinated, challenged, 10 days post-infection.

Caecal tissue metabolomic profiling was performed for the same chickens as described above using samples collected in parallel with those used for microbiome sequencing analysis to characterize host physiological responses. An untargeted metabolomics approach was applied for screening metabolites within the tissues. Based on Euclidean distance measurements, PCA of caecal tissue metabolome profiles showed a similar pattern to the caecal microbiota with unchallenged and challenged individuals differentiated along the PC1 axis (52.73% of observed variation) (Fig. 3). The recovering (UV-C10) group displayed a broad but intermediate metabolome profile to that of 6 dpi challenged chickens and uninfected chickens, and this group was also differentiated along the PC2 axis (9.57%). The values on the PC1 correlated with caecal lesion scores (|*r*| = 0.83, *P* < 0.001) and *Eimeria* loads (qPCR ratio: |*r*| = 0.73, *P* < 0.001; NGS reads of caecal contents: |*r*| = 0.83, *P* < 0.001; NGS reads of caecal tissues: |*r*| = 0.68, *P* < 0.001) (Fig. 3B and C). Among 1,180 metabolites belonging to the 10 categories that were detected from all chickens (including partially characterized and uncharacterized; Fig. 3D), 954 metabolites were either negatively (606, non-infection-associated) or positively (348, infection-associated) correlated with pathophysiology changes (lesion scores and *Eimeria* loads; significant negative correlation with PC1 in Fig. 3A by Pearson’s correlation analysis, FDR < 0.1; Fig. 3E). In more detail, xenobiotics, cofactors and vitamins, especially vitamin Bs, were characterized as non-infection-associated metabolites (Fig. S2A; Table S2); while lipids, especially the sphingolipids, nucleotides, and carbohydrates, were characterized as infection-associated metabolites (Fig. S2B; Table S2).

### Multi-omics factor analysis reveals covariation patterns of disease status

**Fig 4:**
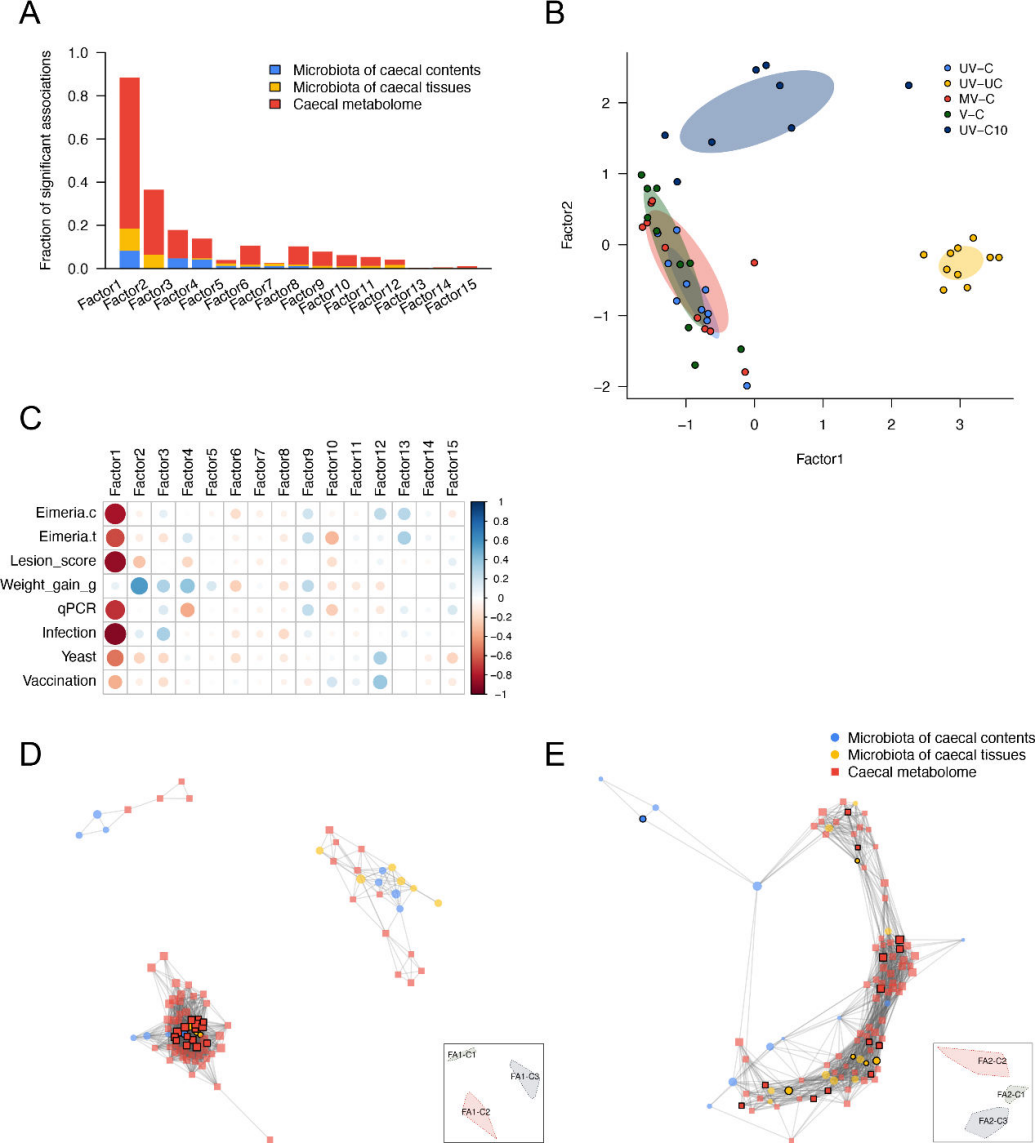
MOFA model for all trial groups and downstream signature marker identification by network analysis. (**A**) Bar plots showing the fraction of significant associations between the features of each microbiome or metabolome modality and each factor. The stacked bars interpret whether the variance-explained values are driven by a strong change in a small number of features or by a moderate effect across a large range of features. (**B**) Scatterplot of factor 1 (*x* axis) versus factor 2 (*y* axis). Each dot represents a sample, colored by the trial group. (**C**) The correlation heatmap of MOFA factors and phenotypes (Eimeria.c: NGS read-based *Eimeria* load in caecal contents; Eimeria.t: NGS read-based *Eimeria* load in caecal tissues; qPCR: qPCR-based *Eimeria* load in caecal tissues; Infection: infection condition- infected or non-infected; Yeast: yeast vector exposure or not; Vaccination: vaccination condition- vaccinated or non-vaccinated). (**D**) and (**E**) Network analysis and visualization for the features from (**D**) factor 1 and (**E**) factor 2. The top 20% of hub centrality nodes were highlighted with black frames; the annotations of hub features are shown in Table S3. Thumbnail legends present the regions of subnetworks. Details of subnetworks from the MOFA model can be referred to in Fig. S3.

Using MOFA, integration of parallel caecal tissue and content microbiomes with caecal tissue metabolome data showed concordant responses that associated with gut pathology and parasite load. Host-microbe intercorrelated features were assessed between microbial and metabolite features using Spearman’s correlation. A total of 151 KTUs and 767 metabolites were significantly associated (FDR < 0.05), resulting in an MOFA model that contained 15 representative factors. The factors were decomposed and ordered by the fraction of significant associations they contributed to the major variances (Fig. 4A). The first two MOFA factors explained the most variance that differentiated the unchallenged, challenged, and recovering groups on the MOFA scatter plot (Fig. 4B). In addition, covariate (phenotype) correlation analysis demonstrated that the first two MOFA factors were associated with the majority of the covariates (Fig. 4C), where factor 1 (FA1) was particularly associated with covariates related to infection (*r* < −0.6) and factor 2 (FA2) was associated with BWG (*r* = 0.56); associations not identified in correlations of single omics analyses.

Multi-omics networks can contextualize the multiple types of microbiome disruption associated with various biological molecules found in different health statuses (28). Additionally, a network’s hotspot molecular features (hubs and clusters) can highlight targets to be followed up. Here, we conducted network analyses downstream of MOFA to explore biomarkers that might be associated with anticoccidial vaccination. Network analyses for the MOFA factors showed sub-structures (clusters of intercorrelated features) that were enriched in each MOFA factor (Fig. 4D and E). Three clusters were identified from FA1 components; two were associated with *Eimeria* challenged chickens (including unvaccinated, vaccinated, and recovering groups) (FA1-C1 and C3 in Fig. 4D), while the third was associated exclusively with unchallenged chickens (FA1-C2 in Fig. 4D). Additionally, cluster 1 in the FA2 network demonstrated associations between unchallenged/recovering groups and the 6 dpi challenged group (FA2-C1 in Fig. 4E). Clustered components from the FA1 and FA2 networks associated with non-challenge and recovery were enriched by vitamin B and derivatives (e.g., pyridoxine, riboflavin, and nicotinate derivatives), short-chain fatty acids (e.g., butyrate/isobutyrate and valerate), and short-chain fatty acid-producing bacteria (e.g., *Caproicibacter fermentans* and *Ruminococcoides bili*). Itaconate, an antipathogenic organic acid was enriched in recovering chickens. In contrast, uremic toxin (e.g., p-cresol sulfate), the long-chain fatty acids and derivatives [e.g., 14—18C fatty acids and glycerophospholipids (GPs), glycerophosphocholine (GPC), and phosphoethanolamine (PE) derivatives], metabolites of fatty acid metabolism (eicosenoylcarnitine and docosadienoylcarnitine), and gut pathogens (e.g., *C. difficile* and *C. innocuum*) and commensal bacteria (e.g, *E. coli*, *Clostridium bolteae*, and *Fecalibacterium prausnitzii*), were enriched in post-*Eimeria* challenged associated clusters of both networks (Fig. S3). Hub centrality scoring of the network identified exclusive key features (top 20% high hub centrality nodes). Cellulose/complex carbohydrate-degrading bacterium—*K*. *alysoides*, amino acid utilization bacterium—*Agathobaculum desmolans*, cholate and its secondary bile acids product—ursodeoxycholate, and gut microbial producing isoflavone antioxidant—6-hydroxydaidzein were potential markers of healthy and recovered chickens' gut ecosystem; whereas *C. innocuum* reflected compromise of the gut barrier after infection (Table S3).

### MOFA models discover potential signature markers of host response to challenge after vaccination

**Fig 5:**
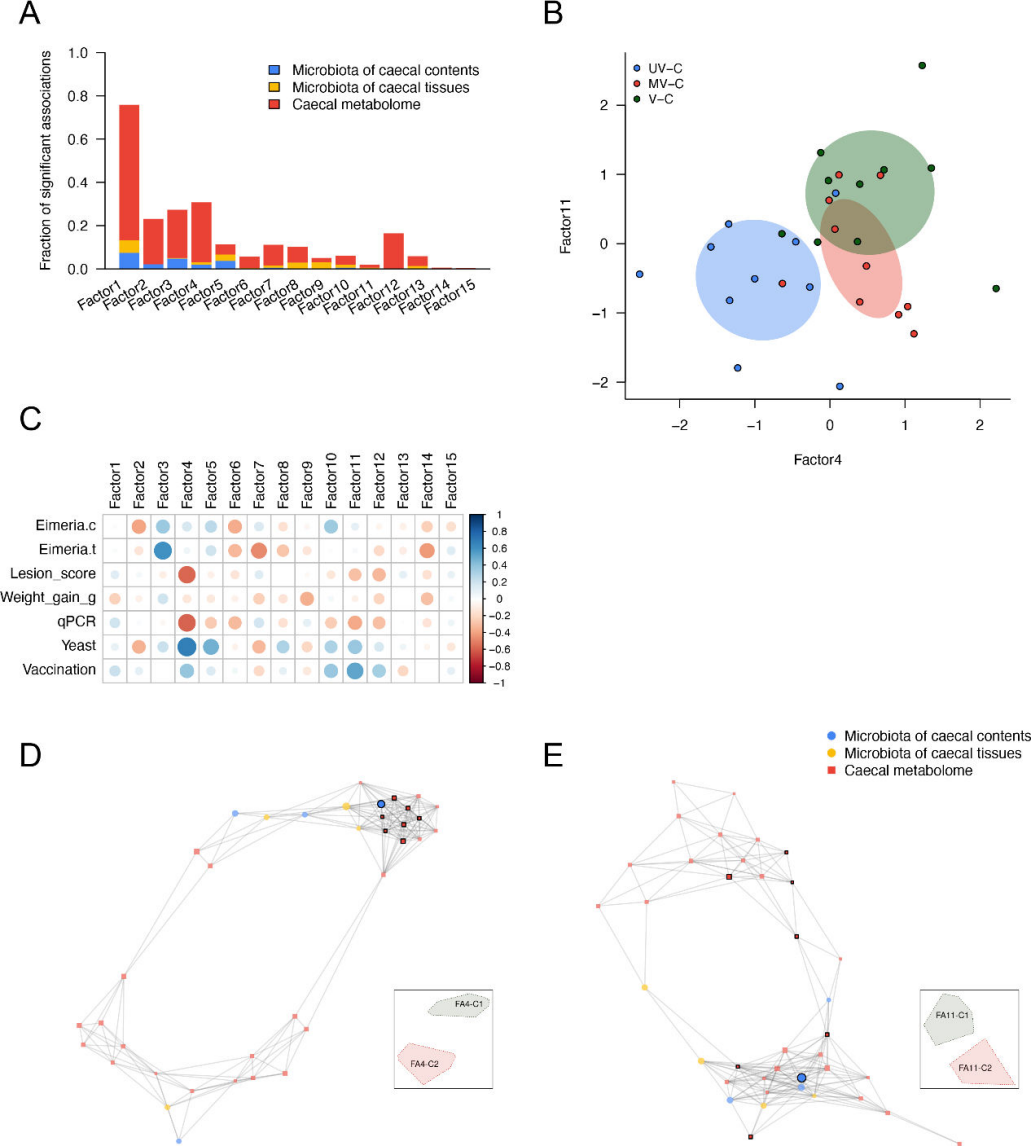
MOFA model for challenged 6 dpi chickens and downstream signature marker identification by network analysis. (**A**) Bar plots showing the fraction of significant associations between the features of each microbiome or metabolome modality and each factor. The stacked bars interpret whether the variance-explained values are driven by a strong change in a small number of features or by a moderate effect across a large range of features. (**B**) Scatterplot of factor 4 (*x* axis) versus factor 11 (*y* axis). Each dot represents a sample, colored by the trial group. (**C**) The correlation heatmap of MOFA factors and phenotypes (Eimeria.c: NGS read-based *Eimeria* load in caecal contents; Eimeria.t: NGS read-based *Eimeria* load in caecal tissues; qPCR: qPCR-based *Eimeria* load in caecal tissues; Yeast: yeast vector exposure or not; Vaccination: vaccination condition- vaccinated or non-vaccinated). (**D**) and (**E**) Network analysis and visualization for the features from (**D**) factor 4 and (**E**) factor 11. The top 20% of hub centrality nodes were highlighted with black frames; the annotations of hub features are shown in Table S4. Thumbnail legends present the regions of subnetworks. Details of subnetworks from the MOFA model can be referred to in Fig. S4.

While highlighting the covariation patterns of disease status, the MOFA model constructed using data from all samples did not reveal factors specifically associated with unvaccinated-challenged and vaccinated-challenged (mock and true vaccines) chickens. A more focused MOFA model was performed on all 6 dpi challenged groups to identify signature markers after vaccination. In the second model, the first four MOFA factors contributed to the major variation of the data and the fraction of significant associations (Fig. 5A). Interestingly, the phenotypic and pathological covariates were more closely associated with FA4 and FA11 (e.g., lesion score severity was more associated with FA4 than other FA; *r* = −0.57). Vaccine treatment conditions (Yeast: treating with yeast vectors or not; Vaccination: treating with the true vaccine or not) were negatively associated with FA4 and FA11, and the parasite load (qPCR ratio) was associated with both FAs (*r* = −0.59 and −0.37) (Fig. 5C). Comparison of FA4 and FA11 using a scatter plot demonstrated that FA4 clearly distinguished the treatment condition of yeast vectors between unvaccinated (UV-C) and vaccinated groups (MV-C and V-C). FA11 showed a different trend between the mock vaccine group (MV-C) and the true vaccine group (V-C) (Fig. 5B). Using network analysis, the signature features of various sphingolipids (e.g, sphingosine and sphingomyelin) and *Ruminococcus lactaris* were clustered from both FAs and enriched in most vaccinated subjects; whereas the long-chain fatty acids (e.g., linoleoyl-arachidonoyl-glycerol and oleoyl-oleoyl-glycerol) were enriched in unvaccinated-unchallenged chickens (Fig. 5D and E; Fig. S4). In addition, the sphingolipids were also identified as key features of vaccination based on network hub centrality scoring (Table S4).

## DISCUSSION

An experimental yeast-vectored anticoccidial vaccine has recently been described as a step towards improved control of *Eimeria* species such as *E. tenella*, which cause coccidiosis in chickens (10). Small-scale studies under commercial conditions found that vaccination could partially control the direct consequences of live parasite challenge, reducing parasite replication and its associated enteric pathology, while protecting performance (BWG and feed conversion ratio). In the present study, we have assessed the impact of vaccination on indirect consequences of *Eimeria* infection including microbial dysbiosis and metabolic disruption.

Using 16S rDNA amplicon sequencing from caecal contents and caecal tissue of experimentally vaccinated and challenged commercial Cobb500 chickens found lower microbiota richness (observed KTUs) than in a recent farm study (27), likely due to a necessary enrofloxacin medication in the early rearing period to control an outbreak of colibacillosis. It is well described that antibiotic treatment influences microbiome composition and richness, often recovering to its normal composition after stopping the treatment (29, 30). While this treatment was unexpected, such necessary treatments are common under the field conditions adopted here and were identical across all treatment groups, permitting valid comparison with real-world relevance. Microbiota richness is also expected to be higher in populations of mixed-breed chickens reared in the field under varied husbandry regimes than under the controlled conditions used in the present study. Comparison between caecal contents and tissues found no significant differences in alpha diversity (*P* = 0.87) or beta diversity (*P* = 0.052) (Fig. S1). Only *Anaerotruncus (A.) colihominis* and *Flavonifractor (F.) plautii* were consistently enriched in caecal tissue samples across multiple groups (UV-UC, MV-C, and V-C), indicating their association with the intestinal mucosal environment. *A. colihominis*, originally isolated from mouse colonic mucosa by the Leibniz Institute DSMZ, has been detected in the intestinal lumen and stool samples of patients with bacteraemia and colorectal cancer, suggesting a potential broader role in gut dysbiosis and pathology (31, 32). Similarly, *F. plautii*, known for its ability to degrade flavonoids and potentially mucins, was isolated by Levine et al. from mammalian intestinal mucosa (33). Its presence in these tissue samples underscores its importance in gut health and disease (33, 34). Based on our findings, investigation of caecal contents alone appears to be sufficient to investigate total gut microbiota because these reflect the primary condition of the intestinal ecosystem.

*Eimeria* infection is known to predispose chickens to diseases such as necrotic enteritis, caused by *C. perfringens* (35), and can disrupt enteric microbial populations leading to dysbiosis (21). We anticipated that beta diversity, but not alpha diversity, would change following *Eimeria* challenge. However, although average richness (observed KTUs) was lower in unchallenged chickens, the difference was not statistically significant (Fig. 2). Comparison of bacterial abundance between infected and non-infected chickens revealed increased Gammaproteobacteria and potentially pathogenic *Clostridia* in *Eimeria*-challenged chickens. Common gastrointestinal pathogens, including *Escherichia (E.) coli*, *Clostridium (C.) difficile*, *Enterococcus (E.) cecorum*, *P. mirabilis*, and *Clostridium (C.) innocuum*, were also in higher abundance (Fig. 2H) suggesting significant dysbiosis occurred following *Eimeria* challenge infection. It is notable that some strains of *E. caecorum* have been reported to cause high morbidity and mortality in broiler chickens (36). Additionally, *C. difficile* and *C. innocuum* can cause antibiotic-associated diarrhea and have shown vancomycin resistance (37, 38), suggesting that a compromised gut environment may facilitate colonization by antibiotic-resistant strains; this condition mirrors the mechanism of human pseudomembranous colitis, which arises due to the overgrowth of *C. difficile* following extensive antibiotic usage. *Eimeria* infection can alter the gut microenvironment by increasing intestinal permeability and inflammation (39), thereby interacting bidirectionally with the gut microbiota. Consequences of enteric dysbiosis include immune dysregulation causing gut-related disorders such as allergies, inflammatory bowel disease and autoimmune disorders (40–42). Thus, *Eimeria* challenge is likely to activate a synergistic response between the host’s physiology and the commensal gut microbiota. Intestinal infections can decrease oxygen levels and lead to chronic tissue and mucosal hypoxia with dysregulation of activation of hypoxia-inducible factors and NF-κB, exacerbating inflammation and injury of intestinal tissues (43, 44). The metabolic environment of the mucosa is also altered during inflammation since the Enterobacteriaceae require terminal electron acceptors from the mucosa for anaerobic respiration and blooming (45, 46). Inflammatory cells release ROS and RNS, forming NO_3_^−^ as a terminal electron acceptor for Gammaproteobacteria growth via denitrification (45, 47–53).

In all *Eimeria*-challenged groups (UV-C, MV-C, and V-C), the gut microbiota composition was similar (Fig. 2A). However, yeast treatment groups (MV-C and V-C) showed a significant increase in *E. coli* abundance, nearly double in UV-C and over six times higher in UV-UC. While harmful *E. coli* may increase due to infection, it is possible that some protective *E. coli* strains that can stimulate an innate immune mechanism (54) and produce vitamins (55, 56) colonize after the reversion of dysbiosis. Notably, *Bifidobacterium*, a common lactic acid-producing probiotic, was present in both yeast treatment groups, irrespective of *Eimeria* antigen expression. In addition, Lactobacillales family bacteria (*Enterococcus*, *Lactobacillus*, and *Pediococcus*) were enriched in both non-infected and yeast treatment groups, with *Lactobacillus* and *Pediococcus* being particularly higher in non-infected groups. This enrichment suggests a beneficial modulation of the gut microbiota. Yeasts and lactic acid-producing bacteria, often found together in nature (57), decrease pH value during fermentation creating an unfavorable environment for some pathogens (58, 59).

We used a multi-omics integrative tool, MOFA, to infer how the caecal metabolome interacts with gut microbes under a range of vaccination and *Eimeria* infection conditions. MOFA modeling confirmed that metabolites involved in fatty acid metabolism and β-oxidation pathways were altered by *Eimeria* infection (23). Inflammation and oxidative stress induced by *Eimeria* invasion and subsequent pathology increase the demand for metabolites involved in fatty acid metabolism (60). The model found that carnitine derivatives such as eicosenolycarnitine and docosadienolycarnitine, intermediate metabolites involved in fatty acid metabolism, were enriched in the *Eimeria* challenged groups (challenge groups compared to non-challenge and recovering groups; factor 2 of MOFA model 1). In addition, p-cresol sulfate (pCS), a uremic toxin formed by gut microbial fermentation of tyrosine (61, 62), was also enriched in all challenged groups, especially in unvaccinated, challenged chickens (factor 1 of MOFA model 1). The main producer of pCS, *C. difficile*, a significant cause of diarrhea during microbial ecosystem collapse, was also identified (factor 1 of MOFA model 1) (63, 64). These findings link both layers of omics and prove evidence that *Eimeria* infection causes dysbiosis.

Since the first MOFA model (the full model with all groups of the trial) could not distinguish an effect of vaccination among the challenged, non-challenged, and recovering groups, a second MOFA model was used to explore latent grouping among vaccinated and non-vaccinated chickens. We found sphingolipids, including sphingosine, sphingomyelin, and sphingoinositol, were significant factors associated with vaccination. Sphingolipids are required in cell membrane structures of eukaryotes (especially the Schwann’s cell, which surrounds the neuron axon) and some prokaryotes (65), as well as essential signaling molecules of inflammatory, immunity, cell autophagy, growth, and survival regulations (65–69). Brown et al. (70) indicated that the microbe-derived sphingolipids (especially from Bacteroides) are negatively correlated with gastrointestinal inflammation (i.e., inflammatory bowel disease) and maintaining homeostasis and symbiosis of gut microbiota (70). This finding supports the efficacy of the yeast-based oral anti-coccidiosis vaccine and indicates that the vaccine can alter the symbiosis status of gut microbiota. However, only a few reads of Bacteroides were detected from yeast-based vaccine-treated samples and non-*Eimeria*-challenged samples (including from caecal tissues and contents), possibly due to the early antibiotic treatment of all study subjects. It implies that the microbial anti-inflammatory sphingolipids could be produced via other microbial species in the chicken gut microbiota, then act as a signal of anti-coccidiosis for further applications.

In conclusion, using MOFA machine learning to integrate evaluation of potential interactions between the enteric microbiome and host-microbe interacted metabolism provided a mechanistic insight into the effects of anticoccidial vaccination and *Eimeria* challenge. In the present study, we identified Gamma-proteobacteria, *p*-cresol sulfate, *Bifidobacterium*, carnitine-derived metabolites, and sphingolipids as host-microbe-associated biomarkers that vary between healthy, infected, vaccinated, and/or recovering chickens, providing insights into potential strategies for controlling, treating, and preventing coccidiosis. As we look to the future, the findings of this study are poised to contribute to the advancement of precision agriculture, particularly in enhancing poultry health management and the development of novel interventions against coccidiosis.

## MATERIALS AND METHODS

### Study animals, metadata measurement, and study design

Cobb500 broiler chickens were purchased from P. D. Hook (Hatcheries) Ltd. (Cote, UK) at day of hatch and reared under commercial conditions. All chickens received enrofloxacin (Baytril, Bayer, Leverkusen, Germany, 10 mg kg^−1^) from days 16 to 18 of the trial due to an outbreak of colibacillosis. All groups received the same treatment, an intervention common in commercial production systems. Feeding and vaccination treatments were as described in a previous study (Study 4 in reference (10)). Briefly, four groups of ten chickens were sampled from a larger vaccination study 6 days post*-E. tenella* challenge including (1) unvaccinated, challenged (UV-C) (2), unvaccinated, unchallenged (UV-UC) (3), mock vaccinated, challenged (MV-C), and (4) vaccinated, challenged (V-C) groups. A fifth group of eight unvaccinated, challenged chickens were sampled 10 days post-challenge (UV-C10; Table S1). Mock and experimental vaccines were administered by oral inoculation in 100 µL phosphate-buffered saline every 3–4 days from day 7 of age (five doses per chicken in total). Group 3 (MV-C) was vaccinated using a mock vaccine including *S. cerevisiae* EBY100 strain (Invitrogen, Thermofisher Scientific, Waltham, MA, USA) containing the empty yeast display plasmid vector pYD1 (Invitrogen). Group 4 (V-C) was vaccinated at the same time points by oral inoculation of an experimental trivalent formulation of *S. cerevisiae*-vectored recombinant vaccine using pYD1 to separately express each of three *E. tenella* antigens including EtAMA1 ectodomain (11), EtIMP1 (12), and EtMIC3 (13). The vaccine design and administration procedures were as described previously (10). Groups 1, 3, 4, and 5 were challenged by oral inoculation with 15,000 sporulated *E. tenella* Houghton strain oocysts at 21 days of age. Challenge oocysts were prepared and inoculated following established protocols (71). Caeca (paired) were collected immediately post-mortem at 6 or 10 dpi (Groups 1–4, and 5, respectively). The severity of infection was assessed using the Johnson and Reid scoring system (72). Overall production performance was defined by BWG between 0 and 6 dpi. Parasite replication was measured using quantitative PCR for parasite genomes per host genome (10); and *Eimeria* apicoplast 16S rDNA, identified by the SILVA database (v138 database collection ID: CBUU010051530.238.1796), was captured by NGS sequencing and used to quantify parasite loads.

### DNA extraction and 16S rDNA amplicon sequencing

Bacterial genomic DNA was extracted separately from caecal tissue (~100 mg) and caecal contents (~200  mg) using a QIAamp Fast DNA Stool Mini kit (QIAGEN, Valencia, CA, USA) following the manufacturer’s pathogen detection protocol. 16S rDNA amplicon library preparation followed the Illumina 16S Metagenomic Sequencing Library Preparation guidelines (73). The 16SrDNA V3–V4 hypervariable regions were amplified by PCR with the adapter overhang primers 341F (5′-TCGTCGGCAGCGTCAGATGTGTATAAGAGACAGC CCTACGGGNGGCWGCAG-3′) and 805R (5′-GTCTCGTGGGCTCGGAGATGTGTATAAGAGACAG GACTACHVGGGTATCTAATCC-3′) for 25 cycles. Indices and Illumina sequencing adapters were attached using the Nextera XT Index Kit with eight cycles of a second amplification reaction. The final PCR products were purified using AMPure XP beads (Beckman Coulter, Brea, CA, USA). The amplicon DNA concentration was measured using Qubit dsDNA *HS* and *BR* Assay Kits (Thermo Fisher Scientific, Waltham, MA, USA). Library quality was determined using the Agilent Technologies 2100 Bioanalyzer system with a DNA-1000 chip. Eighty-eight samples representing caecal tissues from all chickens in Groups 1–5 (*n* = 48) and caecal contents from all chickens in Groups 1–4 (*n* = 40) were pooled with equal molality. The 16S rDNA amplicon libraries were sequenced using a 301-bp paired-end (301 bp × 2) approach on an Illumina MiSeq platform using V3 chemistry.

### Bioinformatic processing and microbiota analyses

The Illumina MiSeq platform generated a total of 22,525,182 paired-end sequences. Sequences were cleaned by sequence length ≥300 bp using Trimmomatic (74). The 16S rDNA amplicon sequences were processed using the Quantitative Insights Into Microbial Ecology 2 (QIIME 2) pipeline (version 2019.10) (75). Primer sequences were removed by Cutadapt (version 1.15) (76). Trimmed sequences were truncated at 240 bp (forward) and 210 (reverse) and denoised using the DADA2 algorithm (77). Amplicon sequence variants (ASVs) were obtained via the denoising process with quality filtering and chimera removal. A k-mer based re-clustering algorithm “KTU” (78) was subsequently applied to assemble ASVs into optimal biological taxonomic units (KTUs). KTUs taxonomy was assigned by comparison with the SILVA SSU reference nr99 (v138) (79, 80) and NCBI 16S RefSeq (retrieved 10 February 2022) databases using the taxonomy function of the KTU R-package. Eukaryotic organelle 16S sequences (identified as *Eimeria*) were extracted and used for supplementary parasite load quantification; non-prokaryotic and unassigned KTUs were removed from the microbiota data set. The 309 KTU microbiota data set was rarefied at the minimum read counts among samples (10,034 reads) after removing twelve samples with shallow sequence depth (<10,000 reads).

Microbiota analyses were conducted and visualized using the Microbiome Analysis R code (MARco) (81), Community Ecology “vegan” (82), and Pretty Heatmap (pheatmap) (83) packages in R (version 4.0.1) (84). The ANOVA test with Tukey HSD *post hoc* multiple comparison test or Kruskal-Wallis test with Dunn’s *post hoc* multiple comparison test were used for parametric and non-parametric statistical analyses of group comparisons with a significance level of *α* = 0.05, and the *P* values were adjusted with an FDR. Alpha diversity indices were estimated by richness. Beta diversity of microbial communities was measured by Bray-Curtis dissimilarity using PCoA, and heterogeneity was tested using ADONIS and ANOSIM tests.

### Metabolome profiling

Untargeted metabolome profiling of caecal tissues was performed by Metabolon (NC, USA) using their vendor protocol. Briefly, all samples were deproteinized by dissociating small molecules bound to protein or trapped in the precipitated protein matrix. To recover chemically diverse metabolites, methanol was used for protein precipitation under vigorous shaking for 2 min (Glen Mills GenoGrinder 2000), followed by centrifugation. The extract was aliquoted into five fractions: two for analysis by separate reverse phase (RP)/UPLC-MS/MS methods with positive ion mode electrospray ionization (ESI), one for analysis by RP/UPLC-MS/MS with negative ion mode ESI, one for analysis by HILIC/UPLC-MS/MS with negative ion mode ESI, and one sample was reserved as backup. Samples were placed briefly on a TurboVap (Zymark) to remove the organic solvent. The sample extracts were stored overnight under nitrogen before preparation for analysis.

All methods used Waters ACQUITY ultra-performance liquid chromatography (UPLC) and a Thermo Scientific Q-Exactive high resolution/accurate mass spectrometer interfaced with a heated electrospray ionization (HESI-II) source and Orbitrap mass analyzer operated at 35,000 mass resolution. Each sample extract was dried then reconstituted in solvents compatible with each of the four methods. Each reconstitution solvent contained a series of standards at fixed concentrations to ensure injection and chromatographic consistency. One aliquot was analyzed using acidic positive ion conditions, chromatographically optimized for more hydrophilic compounds. The extract was gradient eluted from a C18 column (Waters UPLC BEH C18—2.1 × 100 mm^2^, 1.7 µm) using water and methanol, containing 0.05% perfluoropentanoic acid (PFPA) and 0.1% formic acid (FA). Another aliquot was also analyzed using acidic positive ion conditions; however, it was chromatographically optimized for more hydrophobic compounds. The extract was gradient eluted from the same aforementioned C18 column using methanol, acetonitrile, water, 0.05% PFPA and 0.01% FA and was operated at an overall higher organic content. Another aliquot was analyzed using basic negative ion optimized conditions using a separate dedicated C18 column. The basic extracts were gradient eluted from the column using methanol and water, however with 6.5 mM ammonium bicarbonate at pH 8. The fourth aliquot was analyzed via negative ionization following elution from a HILIC column (Waters UPLC BEH Amide 2.1 × 150 mm^2^, 1.7 µm) using a gradient consisting of water and acetonitrile with 10 mM ammonium formate, pH 10.8. The MS analysis alternated between MS and data-dependent MS^*n*^ scans using dynamic exclusion. The scan range varied slightly between methods, but covered 70–1,000 *m/z*.

Raw data were extracted, peak-identified and QC processed by Metabolon’s in-house systems. Compounds were identified by comparison to library entries of purified standards or recurrent unknown entities. The in-house library was built and maintained by Metabolon, and contained more than 3,300 commercially available purified standard compounds with the information of retention time/index (RI), mass-to-charge ratio (*m/z*), and chromatographic data (including MS/MS spectral data). Compound identification was based on the following criteria: retention index within a narrow RI window of the proposed identification, accurate mass match to the library ±10 ppm, and the MS/MS forward and reverse scores between the experimental data and authentic standards. The identified compounds were categorized into 10 groups (amino acid, carbohydrate, lipid, etc.), which were labeled as super pathways in Metabolon’s data report.

A subset of 1,180 metabolites was detected from the untargeted metabolomics screen. Each metabolite’s peak area (i.e. total ion counts, integrated area under the curve) was median-scaled to normalize. The missing values were then imputed with the observed minimum of each metabolite. Since the metabolomic data were typically close to log-normal distribution, the normalized-imputed data were transformed using the natural log for subsequent analyses.

### MOFA model for microbiota and metabolome integrative analysis

MOFA model fittings were performed to integrate multi-omics data modalities based on an unsupervised machine learning model formulated in a probabilistic Bayesian framework. The 16S rDNA amplicons of caecal tissue and content, and host caecal metabolome were the separate data modalities in this study. In order to make all omics data comparable, the amplicon abundance was centered log-ratio transformed using the “clr” function of the compositions R-package. Spearman’s correlation (FDR < 0.05) was implemented to select associated features from the omics data sets (85). Downstream characterization was performed by variance decomposition, detecting the fraction of significant associations between the features and each factor using Pearson’s correlation (FDR < 0.1), and correlation of phenotype covariates. A sub-grouped MOFA model fitting was performed on all 6 dpi challenged groups. A network analysis for identifying sub-structures of MOFA factors was performed with the R package igraph47 (86). An adjacency matrix based on Spearman’s correlation coefficients of intercorrelated features was constructed from a MOFA factor of interest; these coefficients were also used for assessing length of edges on the network. The latter was conducted with the fast greedy modularity optimization algorithm (87) to identify clusters in the network. The node centrality scores of the network were calculated using the Kleinberg’s hub centrality scores, which were based on the principal eigenvector of the adjacency matrix (88).

## Data Availability

The data set presented in the study is made publicly available. The sequencing data can be accessed at NCBI under BioProject accession number: PRJNA990995. The metabolome data can be accessed at Zenodo under the DOI number: 10.5281/zenodo.12717635.

## SUPPLEMENTAL MATERIAL

Supplemental Material msystems.00947-24-s0001.pdf Supplemental figures and tables.

Table S2 msystems.00947-24-s0002.xlsx Infection-associated metabolites.

ASM does not own the copyrights to Supplemental Material that may be linked to, or accessed through, an article. The authors have granted ASM a non-exclusive, world-wide license to publish the Supplemental Material files. Please contact the corresponding author directly for reuse.
