# Supplementary material for: Multi-omics analysis reveals regime shifts in the gastrointestinal ecosystem in chickens following anticoccidial vaccination and Eimeria tenella challenge
Source: mSystems. 2024 Sep 17;9(10):e00947-24. doi: 10.1128/msystems.00947-24 (PMC11494932; doi:10.1128/msystems.00947-24)
Supplement: Supplemental Material — Supplemental figures and tables. [file msystems.00947-24-s0001.pdf]

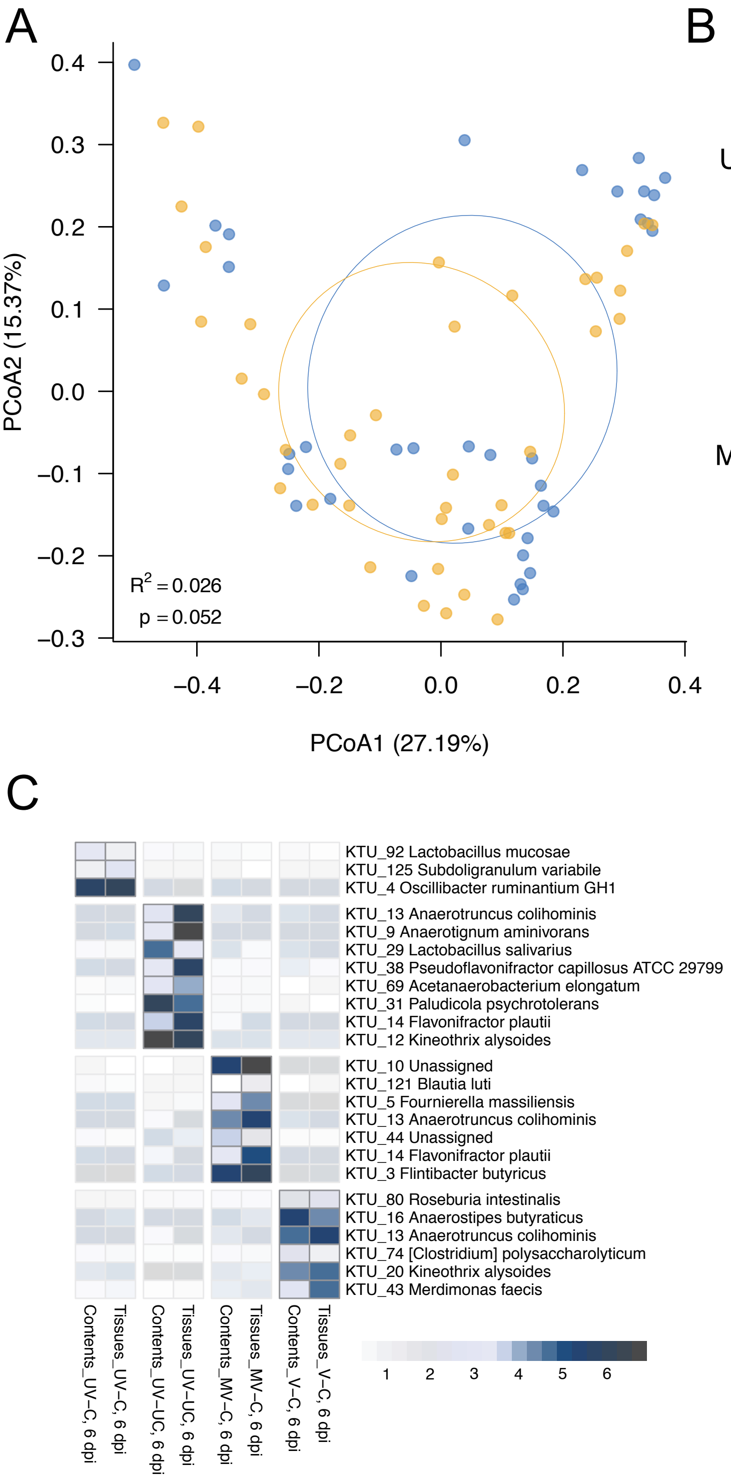


**Figure S1. Comparisons of microbiota profiles between chicken caecal contents and caecal tissues.** (A) Beta diversity analysis with the PERMANOVA test demonstrateed no significant differences between caecal contents and caecal tissues (p=0.052). (B) Venn diagrams show 62.7-73.6% microbiota composition shared between caecal contents and tissues in four study groups (UV-C, UV-UC, MV-C, and V-C). (C) Differential abundance ($p<0.05$ by DESeq2 test) of microbes between caecal contents and caecal tissues in four study groups, respectively.

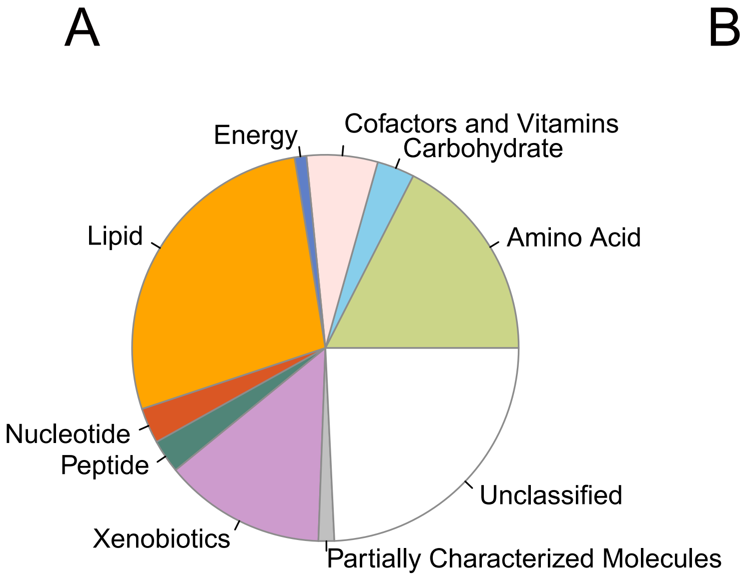


**Figure S2. Lesion score and *Eimeria* load correlated metabolites.** (A) Categories of non-infection-associated metabolites. (B) Categories of infection-associated metabolites.

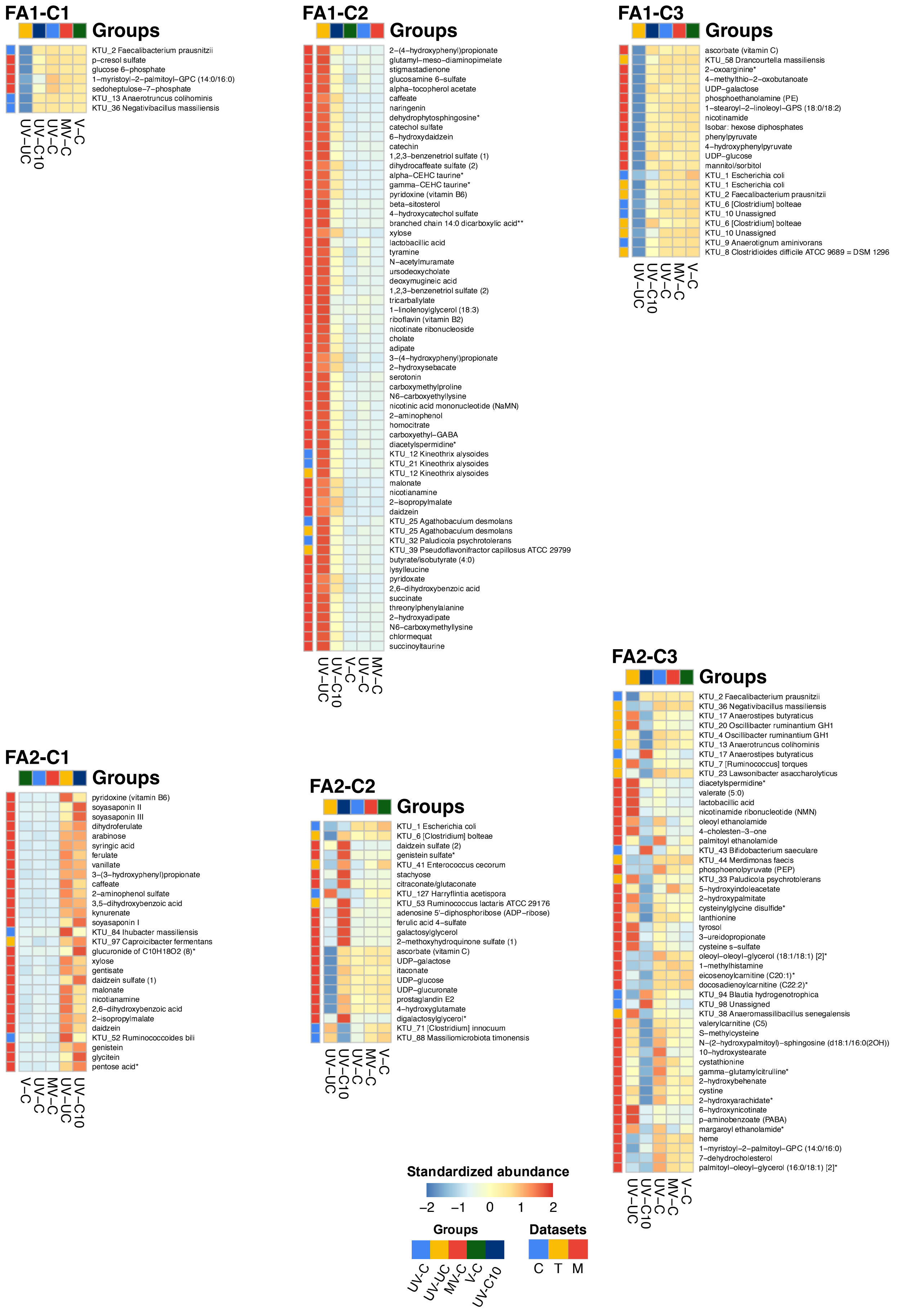


**Figure S3. Details of subnetworks from the MOFA model.** Microbial and metabolomic features of each cluster (C1-C3) in FA1(Figure 4D) and FA2(Figure 4E)—the abundance of microbial and metabolomic features among groups were presented by heatmap. The left columns were annotated by sample sources (C: caecal content microbiome; T: caecal tissue microbiome; M: caecal tissue metabolite).

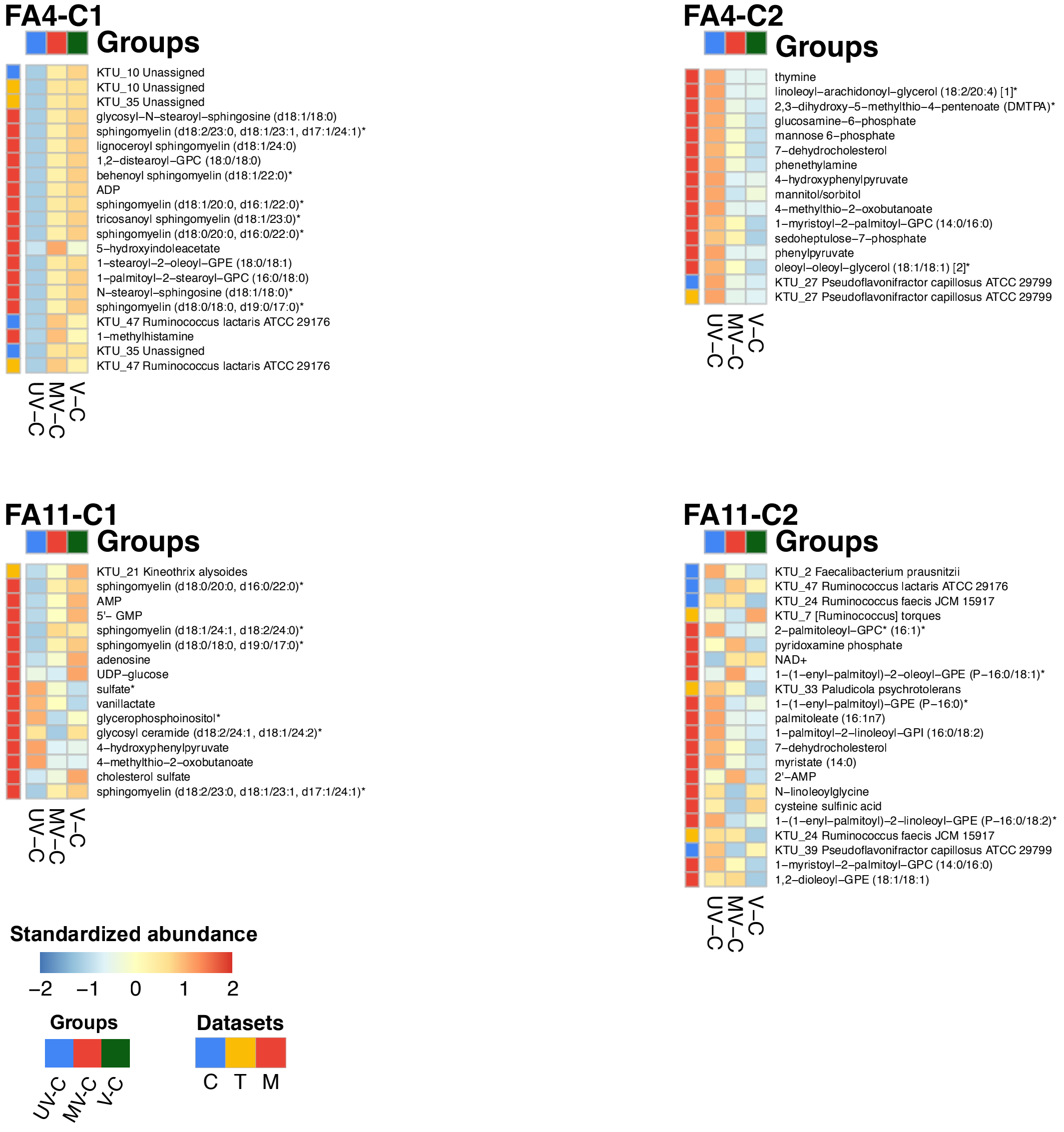


**Figure S4. Details of subnetworks from 6dpi -challenged MOFA model.** Microbial and metabolomic features of each cluster (C1-C2) in FA4(Figure 5D) and FA11(Figure 5E)—the abundance of microbial and metabolomic features among groups were presented by heatmap. The left columns were annotated by sample sources (C: caecal content microbiome; T: caecal tissue microbiome; M: caecal tissue metabolite).

**Table S1. Sample information of studying groups**

| Groups | # of samples | Description | Content ID | Tissue ID |
|---|---|---|---|---|
| UV-C | 10 | Unvaccinated, challenged, 6 dpi | C1 | T1 |
| UV-UC | 10 | Unvaccinated, unchallenged, 6 dpi | C2 | T2 |
| MV-C | 10 | Mock vaccinated, challenged, 6 dpi | C3 | T3 |
| V-C | 10 | Vaccinated, challenged, 6 dpi | C4 | T4 |
| UV-C10 | 8 | Unvaccinated, challenged, 10 dpi | - | T5 |

**Table S3. Top 20% of hub centrality nodes in the full MOFA model networks**

| MOFA model | Factor | Subnetwork cluster | Kleinberg's hub centrality scores | Node annotation | Sample source |
|---|---|---|---|---|---|
| Full model | FA1 | FA1-C2 | 1.000 | dehydrophytosphingosine* | Metabolome |
| | | FA1-C2 | 0.984 | cholate | Metabolome |
| | | FA1-C2 | 0.977 | KTU_12 Kineothrix alysoides | Tissue microbiota |
| | | FA1-C2 | 0.976 | KTU_12 Kineothrix alysoides | Caecal microbiota |
| | | FA1-C2 | 0.973 | 6-hydroxydaidzein | Metabolome |
| | | FA1-C2 | 0.947 | alpha-tocopherol acetate | Metabolome |
| | | FA1-C2 | 0.944 | 1,2,3-benzenetriol sulfate (2) | Metabolome |
| | | FA1-C2 | 0.943 | KTU_39 Pseudoflavonifractor capillosus ATCC 29799 | Tissue microbiota |
| | | FA1-C2 | 0.942 | adipate | Metabolome |
| | | FA1-C2 | 0.939 | 1,2,3-benzenetriol sulfate (1) | Metabolome |
| | | FA1-C2 | 0.939 | nicotianamine | Metabolome |
| | | FA1-C2 | 0.934 | succinate | Metabolome |
| | | FA1-C2 | 0.932 | ursodeoxycholate | Metabolome |
| | | FA1-C2 | 0.928 | carboxymethylproline | Metabolome |
| | | FA1-C2 | 0.913 | KTU_25 Agathobaculum desmolans | Tissue microbiota |
| | | FA1-C2 | 0.913 | 3-(4-hydroxyphenyl)propionate | Metabolome |
| | | FA1-C2 | 0.907 | stigmastadienone | Metabolome |
| | | FA1-C2 | 0.906 | 2-aminophenol | Metabolome |
| | | FA1-C2 | 0.896 | homocitrate | Metabolome |
| Full model | FA2 | FA2-C3 | 1.000 | eicosenoylcarnitine (C20:1)* | Metabolome |
| | | FA2-C3 | 0.984 | KTU_38 Anaeromassilibacillus senegalensis | Tissue microbiota |
| | | FA2-C3 | 0.977 | KTU_20 Oscillibacter ruminantium GH1 | Tissue microbiota |
| | | FA2-C2 | 0.976 | KTU_71 [Clostridium] innocuum | Caecal microbiota |
| | | FA2-C1 | 0.973 | vanillate | Metabolome |
| | | FA2-C3 | 0.947 | 4-cholesten-3-one | Metabolome |
| | | FA2-C1 | 0.944 | soyasaponin II | Metabolome |
| | | FA2-C3 | 0.943 | KTU_33 Paludicola psychrotolerans | Tissue microbiota |
| | | FA2-C3 | 0.942 | nicotinamide ribonucleotide (NMN) | Metabolome |
| | | FA2-C1 | 0.939 | soyasaponin I | Metabolome |
| | | FA2-C3 | 0.939 | lanthionine | Metabolome |
| | | FA2-C2 | 0.934 | KTU_53 Ruminococcus lactaris ATCC 29176 | Tissue microbiota |
| | | FA2-C1 | 0.932 | malonate | Metabolome |
| | | FA2-C3 | 0.928 | oleoyl-oleoyl-glycerol (18:1/18:1) [2]* | Metabolome |
| | | FA2-C3 | 0.913 | KTU_23 Lawsonibacter asaccharolyticus | Tissue microbiota |
| | | FA2-C2 | 0.913 | galactosylglycerol | Metabolome |
| | | FA2-C3 | 0.907 | 2-hydroxybehenate | Metabolome |
| | | FA2-C3 | 0.906 | cysteinylglycine disulfide* | Metabolome |
| | | FA2-C2 | 0.896 | 4-hydroxyglutamate | Metabolome |

**Table S4. Top 20% of hub centrality nodes in the 6dpi-challenged MOFA model networks**

| MOFA model | Factor | Subnetwork cluster | Kleinberg's hub centrality scores | Node annotation | Sample source |
|---|---|---|---|---|---|
| 6dpi-challenged model | FA4 | FA4-C1 | 1.000 | N-stearoyl-sphingosine (d18:1/18:0)* | Metabolome |
| | | FA4-C1 | 0.998 | glycosyl-N-stearoyl-sphingosine (d18:1/18:0) | Metabolome |
| | | FA4-C1 | 0.994 | behenoyl sphingomyelin (d18:1/22:0)* | Metabolome |
| | | FA4-C1 | 0.993 | lignoceroyl sphingomyelin (d18:1/24:0) | Metabolome |
| | | FA4-C1 | 0.989 | sphingomyelin (d18:1/20:0, d16:1/22:0)* | Metabolome |
| | | FA4-C1 | 0.985 | 1-stearoyl-2-oleoyl-GPE (18:0/18:1) | Metabolome |
| | | FA4-C1 | 0.971 | tricosanoyl sphingomyelin (d18:1/23:0)* | Metabolome |
| | | FA4-C1 | 0.970 | KTU_10 Unassigned (Oscillospiraceae spp.) | Caecal microbiota |
| 6dpi-challenged model | FA11 | FA11-C2 | 1.000 | NAD+ | Metabolome |
| | | FA11-C2 | 0.998 | 2'-AMP | Metabolome |
| | | FA11-C2 | 0.994 | 1-(1-enyl-palmitoyl)-GPE (P-16:0)* | Metabolome |
| | | FA11-C1 | 0.993 | 4-methylthio-2-oxobutanoate | Metabolome |
| | | FA11-C1 | 0.989 | sulfate* | Metabolome |
| | | FA11-C1 | 0.985 | glycerophosphoinositol* | Metabolome |
| | | FA11-C1 | 0.971 | vanillactate | Metabolome |
| | | FA11-C2 | 0.970 | KTU_2 Faecalibacterium prausnitzii | Caecal microbiota |
